# Epidemiology of Fungal Bloodstream Infections and Antifungal Susceptibility in a Tertiary Care Hospital in Riyadh, Saudi Arabia: A Rare *Candida* Co-Infection Case

**DOI:** 10.3390/pathogens14121221

**Published:** 2025-11-30

**Authors:** Saeed S. Banawas

**Affiliations:** 1 Department of Medical Laboratory Sciences, College of Applied Medical Sciences, Majmaah University, Al-Majmaah 11952, Saudi Arabia; s.banawas@mu.edu.sa; Tel.: +966-50-870-6523; 2 Health and Basic Science Research Centre, Majmaah University, Al-Majmaah 11952, Saudi Arabia

**Keywords:** fungal infections, non-albicans *Candida*, multi-azole-resistant, epidemiology, tertiary care hospital

## Abstract

Background: In Saudi Arabia, rising multi-drug-resistant (MDR) fungal infections from broad-spectrum antifungal overuse highlight the urgent need for epidemiological and susceptibility research. Methods: This cross-sectional study analyzed fungal isolates from 55 patients with positive blood cultures in a Riyadh tertiary hospital, with identification and antifungal susceptibility tested via the VITEK-2 compact system. Results: *Candida albicans* and non-albicans *Candida* (NAC) were isolated from 11 and 38 patients, respectively. In the NAC group, *C. glabrata* and *C. parapsilosis* spp. were predominant. *C. glabrata* exhibited the highest resistance to antifungals. Increased rates of resistance were shown by fluconazole and itraconazole, whereas voriconazole was the most effective azole with the lowest resistance. No evidence of resistance was found against non-azole antifungals. A single case of triple resistance to ketoconazole, fluconazole, and itraconazole was observed in *C. parapsilosis*. A single isolate of *C. albicans* was resistant to all tested azoles. A rare instance of coinfection with *C. glabrata* and *C. albicans* was identified in a single male patient with a dual-resistance pattern against posaconazole and itraconazole. Conclusions: The high prevalence of NAC, including tolerant isolates of *C. parapsilosis* and *C. glabrata*, along with multi-azole-resistant *C. albicans* and unique coinfection scenarios, urgently requires robust antifungal resistance surveillance.

## 1. Introduction

Fungi are ubiquitous and some species have evolved into opportunistic pathogens that cause diseases in humans, animals, and plants. Fungal infections (FIs), referred to as mycoses, cause a wide range of ailments, from minor skin disorders to severe, potentially fatal situations like bloodstream infections [1]. Once considered rare, FIs have gained global prominence owing to their rising incidence, emergence of drug-resistant strains, and high mortality rates associated with certain fungal diseases. Globally, over one billion individuals have fungal diseases, and approximately 150 million have severe FIs, profoundly impacting their lives [2]. The mortality rate of fungal diseases is estimated to be approximately 1.7 million annually, which is comparable to that of high-risk diseases such as tuberculosis and more than three-fold that of malaria [3,4].

Multiple studies have described the epidemiology of FIs in the Middle East region [5,6,7,8,9,10,11]. Among all nations, Qatar reports highest candidemia (a serious bloodstream infection caused by *Candida* species) incidence rate of 15.4 per 100,000 population [8]. In the UAE, the annual incidence of candidemia is 11.8 per 100,000, with 49.2% of cases occurring in intensive care units (ICUs) [5]. In Turkey, numerous reports have shown that nosocomial candidiasis, including candidemia, ranged between 1.2–5.6 per 1000 admissions [9,10,11]. In Kuwait, candidemia frequency has declined from 0.24 cases in 2014 to 0.15 cases per 1000 patient-days in 2016 [12]. In Kingdon of Saudi Arabia (KSA), the rate of invasive candidiasis ranged from 1.55–1.65 cases per 1000 discharges to 26 cases per 1000 ICU admissions [13,14], reflecting a global trend of rising fungal bloodstream infections in healthcare settings.

Studies have shown that *Candida* genus encompassing a cosmopolitan group of pathogens are the most recurrently isolated micro-organisms in clinical settings [15]. The majority (>90%) of these diseases are caused by five isolates: *C. albicans*, *C. glabrata*, *C. tropicalis*, *C. parapsilosis*, and *C. krusei* [16]. *C. albicans* is a versatile microbe that can acquire tolerance to antifungal agents after extended treatment, and its antifungal resistance has been globally reported [17]. Although *C. albicans* has historically been the most prevalent species [18,19]; however, there is a notable shift toward non-albicans *Candida* (NAC) species [20,21]. This shift is clinically significant, as NAC species often exhibit higher resistance to commonly used antifungals [22,23]. Managing candidemia, a potentially fatal bloodstream infection with a high fatality rate, is made more difficult by this resistance.

A multitude of factors contribute to the rising prevalence of *Candida* infections in KSA. Prolonged hospitalization, particularly in ICUs, is a major risk factor, as patients requiring mechanical ventilation or central venous catheters are highly susceptible to fungal infections [14,24]. Additionally, the high prevalence of chronic diseases including diabetes, cancer, and renal failure, increases vulnerability to *Candida*-related complications, especially in immunocompromised individuals [6,25]. Overuse of broad-spectrum antibiotics in Saudi hospitals has also been linked to an increase in fungal infections, as these medications disrupt the normal microbial flora, allowing fungal overgrowth [26,27]. Environmental factors, including the hot and humid climate in some regions of KSA, may further facilitate fungal persistence and colonization, influencing the epidemiology of *Candida* infections in the region [26]. A retrospective analysis on invasive candidiasis among pediatric patients identified several risk factors: prematurity of patients (28.7%), low birth weight (32.6%), central venous catheter (45.7%), malignancy (16.3%), immunotherapy (15.5%), and ventilator support (46.5%) [28].

Antifungals that are commonly used in managing systemic, aggressive, and persistent FIs are azoles, allylamines, polyenes, and the newly discovered echinocandins [29]. These antifungal medications interfere with diverse cellular processes that combat FIs. Polyenes (e.g., amphotericin B, AmB) attach to the ergosterol on the fungal membrane, affecting membrane permeability [30]. Azoles function by targeting lanosterol 14α-demethylase to reduce the amount of ergosterol on the fungal surface [31]. Although most azole-based antifungals exhibit fungistatic properties, they also act as fungicidal agents against certain molds. Conversely, echinocandins target 1,3-β-glucan synthase activity, leading to alterations in cell wall structure [32]. However, selecting an appropriate empirical treatment is challenging given the increasing prevalence of NAC isolates [33]. Moreover, antifungal resistance complicates the clinical management of patients with FI, leading to higher mortality, increased healthcare costs, limited treatment options, and nosocomial infections.

Antifungal susceptibility testing is crucial for tracking emerging resistance patterns, particularly in *Candida* species, which pose a growing challenge in Saudi hospitals. NAC species exhibit high fluconazole (FLC) resistance, limiting treatment options. While echinocandins are the first-line treatment, resistance in *C. glabrata* [33] highlights the urgency for continuous monitoring. Regular susceptibility testing and species-level identification are essential for optimizing antifungal management programs. Research focusing on epidemiology and antifungal susceptibility testing is, therefore, fundamental for shaping informed medical policies and clinical guidelines [34]. A common fungal blood infection in intensive care units (ICUs), candidemia, emphasizes the value of epidemiological monitoring in directing empirical treatment. In a tertiary-care hospital in Riyadh, Saudi Arabia, the epidemiology of fungal infections—specifically bloodstream infections caused by *Candida* spp.—as well as the patterns of antifungal susceptibility are evaluated in this study.

## 2. Materials and Methods

### 2.1. Study Setting

This cross-sectional investigation was performed in the Microbiology Department of a tertiary-care hospital in Riyadh, KSA, from October 2021 to April 2022. The study adhered to international ethical standards, with prior approval from the Ethics Committee (IRB number: 21-002E). The bulk of the patients were admitted to medical wards, with a subset from intensive care units (ICUs). A total of 55 samples were collected and meticulously categorized based on patient age and gender. Interestingly, 50 samples were identified as *Candida* spp. Informed consent was obtained and confidentiality was ensured throughout the study process. Further, the participants were assured that all data would be used only for research purposes. Included patients were those of all ages (children, adults, and the elderly) and genders admitted to the hospital during the study period with no prior infection or other diseases. Excluded patients had incomplete medical records or missing data, as well as those who were discharged/transferred out of the ICU before data collection. However, due to the cross-sectional design and poor clinical documentation, detailed parameters such as infection site, presence of sepsis or septic shock, and comprehensive comorbidity profiles (including prior antifungal exposure, total parenteral nutrition, and device use) were not consistently available in the hospital records.

### 2.2. Sample Collection and Processing

Blood samples were meticulously collected from peripheral venous sites under strict aseptic conditions to minimize the risk of contamination. Two separate batches of blood samples, each including 5 mL of blood inoculated into aerobic and anaerobic culture bottles, were obtained from each patient at different time points prior to initiation of antifungal therapy. The use of two separate venipunctures (two sets) enhances the likelihood of pathogen detection and helps distinguish true candidemia from potential contamination [35]. No central or arterial line samples were included. All blood draws were performed under physician’s supervision, ensuring that volumes were clinically safe and within routine diagnostic practice. Moreover, all procedures for blood specimen collection, handling, and processing adhered to the Clinical and Laboratory Standards Institute (CLSI) guidelines for antifungal susceptibility testing and blood culture practices [36].

### 2.3. Microbiological Analysis

All blood culture samples were verified to be sterile for bacterial pathogens prior to identification of *Candida* species. Only samples that yielded growth of *Candida* spp. in at least one set of blood cultures and later confirmed by a sequential positive culture were included in the final analysis. This procedure ensures diagnostic accuracy and exclusion of possible contamination.

### 2.4. Identification and Antimicrobial Susceptibility Testing of Fungal Species

Blood samples were cultured using a Biomerieux BacT/Alert^®^ 3D automated blood culture system (Biomerieux, Craponne, France). Positive culture isolates were further sub-cultured on Blood agar and Sabouraud dextrose agar plates (Biomerieux UK Ltd, Basingstoke United Kingdom) after initial Gram staining of the blood culture broth, which revealed the presence of Gram-positive budding yeast. Gram staining and germ-tube assays were done to confirm the presence of the suspected fungal strains. VITEK 2 kits were used to identify yeast and yeast-like organisms (ID-YST cards) with a VITEK^®^ 2 Compact (Biomerieux, Craponne, France). Finally, antifungal susceptibility testing was executed using AST YS07 Kits (Biomerieux, Craponne, France) on a VITEK^®^ 2 Compact according to the manufacturer’s instructions. The process detects fungal growth in the presence of antimicrobial agents by utilizing modified classic fluorogenic and chromogenic substrates as redox indicators. In brief, the ID broth was injected with pure microbial culture by suspending in 0.45% aqueous NaCl calibrated to a 0.5 McFarland range using a CrystalSpec nephelometer (BD Diagnostics, Franklin Lakes, NJ, USA). A 25 mL aliquot of this suspension was taken for antimicrobial susceptibility testing [37].

Antimicrobial susceptibility was accessed following the Clinical and Laboratory Standards Institute (CLSI) guidelines [38]. A valid classification of the isolates required a score more than 90%; otherwise, no identification was documented. Briefly, samples were diluted in RPMI 1640 medium (Sigma, St. Louis, MO, USA) buffered to pH 7 with 0.165 M (3[N-morpholino] propanesulfonic acid) [MOPS] buffer (Sigma, St. Louis, MO, USA). The final inoculum concentration ranged from 1.0 to 1.5 × 10^3^ cells/mL. The antifungal drugs (Sigma, St. Louis, MO, USA) were at final concentrations of 0.125–64 μg/mL for azoles and 0.015–4 μg/mL for AmB and caspofungin (Sigma, St. Louis, MO, USA). After incubation, MIC endpoints for azoles and the echinocandin were read after 24 h as the lowest drug concentration causing a score-2 turbidity drop, indicating about a 50% reduction in growth compared to the control. For AmB, MIC endpoints are defined as the lowest drug concentration showing no visible growth (score 0). The isolate was deemed resistant if the MIC value was ≥8 µg/mL for FLC, ≥16 µg/mL for Ketoconazole, ≥1 µg/mL for Voriconazole, Itraconazole, Posaconazole, AmB and Caspofungin, per CLSI M27/44S document (2022) [39]. *C. parapsilosis* ATCC^®^ 22019 (ATCC, Manassas, VA, USA), the CLSI-recommended strain, was tested for quality.

### 2.5. Data Analysis

The data were subjected to statistical analysis via the Minitab Lab Manual (Minitab, LLC, Chicago, IL, USA). Pairwise comparisons of resistant vs. non-resistant counts between *Candida* species were performed using Fisher’s exact test (two-sided) for each azole. For each drug, we tested all 15 species-pairs. Values were deemed statistically significant at the 95% confidence interval with *p* < 0.05.

## 3. Results

A total of 55 isolates showed variable degrees of resistance to known antifungals. Of these, 28 were collected from male patients while 27 were collected from female patients, almost an equal proportion. Based on patient age, seven isolates were obtained from children aged < 2, and six patients aged 7–12. Only three isolates were found in teenagers (13–19 years). Most isolates (26) were obtained from adults, while some infection patterns (13) were reported in geriatric patients (>65 years). No antifungal resistance was observed in children aged 2–6. The majority of isolates were obtained from hospital wards (72.7%), with lesser proportions collected from the ICU (27.3%) (Table 1).

### 3.1. Diversity of Fungal Isolates

Fungal infections varied from *Candida* spp. to *Rhodotorula* and *Trichosposron* spp. (Table 1). Among the *Candida* species, three strains were predominant in most infections: *C. albicans*, *C. glabrata*, and *C. parapsilosis. C. albicans* was reported in 11 cases, with an equal number of *C. glabrata* and *C. parapsolisis* while *C. tropicalis* was reported in six cases. Furthermore, *C. haemulonii* and *C. auris* were detected in five and three cases, respectively, and one case each of *C. famata*, *C. rugosa*, and *C. lusitaniae* was reported (Table 1). For *C. albicans,* six infected individuals were male and five were female (Table S1). Similar infection profiling in males (five) and females (six) was reported for *C. glabrata*. Most *C. parapsilosis* infections were detected in males (eight), with two cases occurring in females. Smilalry, four cases of *C. haemulonii* were detected in male patients, whereas only one case was reported in a female patient. *C. tropicalis* was predominantly detected in females (five) with only one case identified in a male patient. A single coinfection of *C. albicans* and *C. glabrata* was revealed in a male patient while all cases of *Trichosposron* and *Rhodotorula* and a single case of *C. rugosa* were reported in females. Lastly, *C. famata* and *C. lusitaniae* were detected only in male patients.

### 3.2. Antifungal Resistance Pattern in Clinical Isolates

Antifungal-resistance patterns were evaluated against known antifungals, including azoles, AmB, and caspofungin. For the azoles, most patients (61.8%) exhibited resistance to FLC, which was distributed equally between males and females (Figure S1, Table 2). This was followed by resistance to itraconazole (22.2%) with identical resistance patterns present in both male and female patients. In case of posaconazole, isolates from 18% of patients demonstrated resistance to this antifungal agent, and the majority of the patients were male. Ketoconazole exhibited a resistance pattern similar to that of posaconazole. Among the 55 patients studied, only five (9%) cases of resistance were found, two in males and three in females. Voriconazole was the most effective azole antifungal with the lowest resistance found. Of the 28 reported cases, only three male (10.7%) patients showed resistance, with two intermediate cases in females. Three patients (two female and one male) exhibited an intermediate resistance pattern to FLC. It was noted that not all isolates of each *Candida* species were tested against every antifungal agent (Table 2).

Across azoles, resistance patterns varied markedly by species. FLC resistance was highest among fungal species tested, driven by *C. glabrata* (90.9%), *C. haemulonii* (100%) and *C. auris* (100%), while *C. lustinae*, *C. rugosa* and *C. famata* remained fully susceptible (Table 3). Itraconazole resistance was lower overall but remained high in *C. glabrata* (>90%). Voriconazole resistance was infrequent, though interpretation is limited by the small number of tested isolates; the highest resistance rate was observed in *C. albicans* (43%). Posaconazole resistance was noted only in *C. glabrata* (100%), whereas ketoconazole resistance remained low overall, with the highest rate of 27% also occurring in *C. glabrata*.

Among the azoles, voriconazole showed excellent overall activity against all identified fungal species (Table 3) with low MIC ranges for most species (0.0156–0.25 µg/mL); although three *C. albicans* isolates demonstrated resistance with MIC_50_ and MIC_90_ values of 4 and 16 µg/mL, respectively. Further, approximately 50% of *C. albicans* isolates were resistant to FLC, reflected by elevated MICs extending to 64 µg/mL, and one multi-azole-resistant isolate exhibited resistance to posaconazole (MIC: 32 µg/mL), ketoconazole (MIC: 16 µg/mL), and itraconazole (MIC: 32 µg/mL). Three isolates of *C. albicans* showed resistance to voriconazole with MIC of 4 µg/mL (Table 4).

*C. glabrata* demonstrated the highest azole resistance burden, with all isolates resistant to FLC (MICs ≥ 32 µg/mL) and high-level itraconazole resistance (MIC: 32 µg/mL). *C. parapsilosis* was the third most resistant species after *C. glabrata* and *C. albicans*; six cases of FLC resistance with MICs up to 32 µg/mL were reported for this *Candida* species. A single isolate of this particular species was resistant to both itraconazole (MIC: 32 µg/mL) and ketoconazole (MIC: 32 µg/mL); while 2 isolates exhibited resistance to both FLC (MIC: 64 µg/mL) and itraconazole (MIC: 32 µg/mL).

All isolates of *C. auris* showed high FLC MICs (16–64 µg/mL), while *C. tropicalis* displayed resistance to both FLC (up to 64 µg/mL) and itraconazole (up to 32 µg/mL). Similarly, *C. haemulonii* exhibited markedly elevated FLC MICs (MIC: 32 µg/mL), and all *R. glutinis* isolates were FLC-resistant (MIC: 32 µg/mL). None of the *C. famata*, *C. lusitaniae*, *C. rugosa*, or *Trichosposron* spp., an opportunistic pathogen, exhibited resistance to any of the tested antifungal agents (Table 4).

Despite widespread azole resistance, all species remained susceptible to AmB, with consistently low MIC values (0.125–0.25 µg/mL). Caspofungin activity was also retained across the cohort; *C. albicans* demonstrated MICs of 0.125–0.5 µg/mL, and *C. glabrata* showed even lower MIC values (0.0625–0.125 µg/mL), with no echinocandin resistance observed in any isolate. For several NAC species, caspofungin MICs were not tested.

Fisher pairwise comparisons showed significant FLC resistance differences between high-resistance species (*C. auris*, *C. glabrata*, *C. parapsilosis*) and low-resistance species (*C. tropicalis*, *C. haemulonii*). *C. glabrata* demonstrated significantly higher itraconazole resistance than *C. albicans*, *C. parapsilosis*, and *C. haemulonii* (all *p* < 0.05). No significant inter-species differences remained for voriconazole, posaconazole, or ketoconazole after correction, likely due to small sample sizes. Overall, the findings highlight substantial interspecies variation and confirm significant azole resistance among *C. glabrata*, *C. auris*, *C. haemulonii*, and *R. glutinis*.

### 3.3. Multi-Azole Resistance Pattern

**Figure 1:**
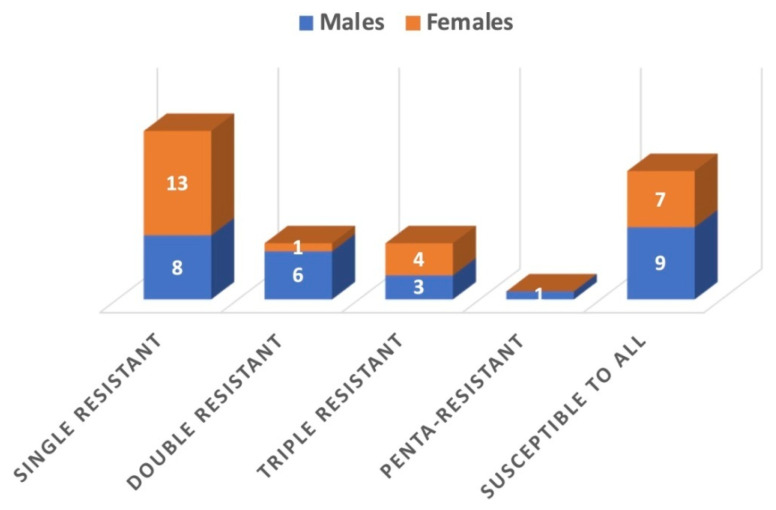
Distribution multi-azole-resistant fungal pathogens among males and females.

In this study, 21 cases of resistance to a single antifungal were noted: 13 cases in female patients and eight in male patients (Figure 1). Among azole antifungals, multiple cases of multi-azole-resistant were reported. Seven confirmed cases of double resistance to two antifungal medications were observed, most of which were reported in males. *C. glabrata* exhibited dual resistance to the combination of itraconazole and FLC as well as to the combination of posaconazole and itraconazole, whereas *C. albicans* was resistant to both voriconazole and FLC. Six cases of triple antifungal resistance patterns were observed with *C. glabrata*: three against a combination of ketoconazole, FLC, and itraconazole, and the rest against a combination of posaconazole, FLC, and itraconazole. A single case of triple resistance to ketoconazole, FLC, and itraconazole was observed in *C. parapsilosis*. One *Candida* species, *C. albicans*, displayed resistance to all tested azole antifungals, raising serious concerns about the management of nosocomial infections (Table 5). FLC and itraconazole were more frequently associated with drug resistance.

### 3.4. An Extraordinary Case of Coinfection with C. glabrata and C. albicans

A rare case of coinfection with two *Candida* spp. (*C. glabrata* and *C. albicans*) was reported in a male patient (Table 6). This coinfected individual displayed a double-antifungal resistance pattern for both fungal species, with *C. albicans* and *C. glabrata*, displaying resistance to posaconazole and itraconazole. As expected, both species were susceptible to the broad-spectrum antifungal agents, AmB and caspofungin. An intermediate reactivity pattern was observed for FLC against *C. glabrata*.

## 4. Discussion

Nosocomial infections pose a severe threat to individuals in tertiary-care hospitals. In Saudi Arabia, invasive candidiasis, particularly *Candida* bloodstream infection (candidemia), remains a significant healthcare concern. Several studies from Saudi Arabia have traditionally identified *C. albicans* as the predominant etiological agent of candidemia in the central [40] and western region [41] of KSA. However, longer-term surveillance data indicate a progressive epidemiological shift toward NAC species, with a seven-year study reporting that although *C. albicans* remained common, NAC isolates formed the majority [42]. This emphasizes the need for diagnostic testing to precisely determine the implicated species to initiate quick and effective therapy.

On a global scale, the five most invasive *Candida* pathogens are *C. albicans, C. glabrata, C. parapsilosis, C. tropicalis,* and *C. krusei* [43,44,45]. Although global SENTRY surveillance continues to put *C. albicans* as the leading bloodstream isolate [46,47], multiple regions are experiencing a marked shift toward NAC species, largely driven by increasing azole resistance. Investigations from the USA, northern Europe, and Australia indicate a pronounced rise in *C. glabrata*, which now ranks the second most prominent cause of candidemia and exhibits reduced antifungal susceptibility [48,49,50,51]. In contrast, countries in tropical Asia, including the Philippines and Thailand, report *C. tropicalis* as the leading NAC species [52,53], consistent with emerging resistance trends [54,55]. Notably, *C. tropicalis* contributes substantially to candidemia worldwide, accounting for 3–66% of cases and associated with 40–70% mortality [56,57]. South Korean data from 2020–2021 also show an epidemiological transition toward *C. glabrata* and *C. tropicalis*, reflecting broader shifts in species distribution [58].

Within Saudi Arabia, species variation exists across cities: in Riyadh, rising NAC infections have been driven by *C. tropicalis* and *C. glabrata* [27], while in Medina, *C. parapsilosis* is most prevalent [24]. Our findings further support this transition, with NAC species (particularly *C. parapsilosis* and *C. glabrata*) predominating (Table 1). Similar distributions have been reported across major tertiary hospitals in KSA: at King Fahad Hospital (Dammam), *C. glabrata* and *C. parapsilosis* were predominant species [7], while a 12-year study from King Faisal Specialist Hospital (Jeddah) found *C. glabrata* to be the leading pathogen (30%), followed by *C. albicans* (23%) [59]. In eastern KSA, at King Fahad Teaching Hospital Al-khobar, *C. parapsilosis* was also the predominant NAC species, followed by *C. tropicalis* and *C. albicans* [60]. These patterns mirror observations from East Asia showing the predominance of *C. parapsilosis* [61], and Middle East regional data, where *C. glabrata* was the most commonly detected pathogen in Lebanese hospitals [22,23]. These regional differences highlight the heterogeneity of *Candida* epidemiology and reinforce the need for localized surveillance and tailored antifungal stewardship strategies.

In addition to the commonly isolated *Candida* species, we identified several rare NAC isolates (Table 2), including *C. haemulonii*, *Candidozyma auris* (formerly *Candida auris*), *C. famata*, *C. rugosa* (recently reclassified as *Diutina rugosa*), and *C. lusitaniae* (Table 1). *C. haemulonii*, an uncommon *Candida* variant, has emerged as an aggressive fungus with increasing clinical relevance due to its reduced susceptibility to several antifungal agents [62]. Similarly, *C. famata*, an atypical subtype, has been implicated in opportunistic fungal diseases, including systemic candidiasis [63]. *C. rugosa* or *D. rugosa* is a newly recognized fungal species affecting both humans and livestock [64]. Meanwhile, *C. lusitaniae* is a rare opportunistic yeast known for its intrinsic resistance to AmB, posing challenges in antifungal therapy [65].

Report from Middle East have documented *C. haemulonii* [66], which is frequently misidentified as *C. auris* [67], although both species exhibit reduced susceptibility to numerous antifungals [66]. A Riyadh tertiary-care study noted a sharp rise in *C. auris* bloodstream infections from 2019 to a peak in 2022, followed by a drop attributed to strengthened infection-control practices [68]. Similarly, the 2024 European Centre for Disease Prevention and Control (ECDC) survey (published 2025) reported >4000 *C. auris* cases across Europe between 2013 and 2023 [69]. These outcomes underline the need for continuous surveillance, rapid diagnostics, and strict infection-control methods to limit the dissemination of *C. auris* and other emerging NAC pathogens.

Antifungal tolerance, particularly to FLC, is of considerable concern to medical practitioners worldwide [70]. A comprehensive SENTRY antimicrobial surveillance system conducted across 22 countries over 2 years revealed that FLC maintained near-perfect (98–100%) efficacy against most *Candida* isolates, except *C. glabrata*, where susceptibility ranged from 48% to 83% [46,47,48,49], consistent with our findings (Table 3). Additional surveillance (2017–2019) documented rising FLC resistance in *C. parapsilosis* [48], and multi-period analyses similarly showed a progressive increase in azole resistance [13]. Our results highlight that resistance patterns are species specific, with *C. albicans*, *C. parapsilosis* and *C. glabrata* showing more extensive resistance to certain drugs, such as FLC and itraconazole, compared to other species like *C. tropicalis* (Table 3 and Table 4). In our study, FLC resistance exceeded 90% in *C. glabrata* and reached 60% in *C. parapsilosis* (Table 3), mirroring regional data from Kuwait and Lebanon [23,24] and matching U.S. trends where FLC resistance in *C. parapsilosis* increased from 8.2% to 20.3% between 2015 and 2024 [71]; thus, posing a significant concern.

The high-level FLC resistance observed in *C. haemulonii* in our study (Table 4) is consistent with reports of universal resistance in the *C. haemulonii* complex during neonatal outbreaks [62]. Another study reported that *C. haemulonii* showed 100% resistance against all antifungals with MIC values ≥ 64 mg/L [72]. Additionally, all three *Rhodotorula glutinis* isolates were FLC-resistant (100%), consistent with reports of very high MICs (>64 mg/L) and intrinsic azole non-susceptibility in this genus [73,74]. Generally, *Rhodotorula* bloodstream infections have lower mortality than those caused by *Candida* or other yeast-like fungi, yet their inherent azole resistance can provide a selective advantage when susceptible fungi are suppressed. Furthermore, *C. tropicalis* demonstrated lower FLC resistance in our study (Table 4), similar to patterns seen in previous research [75]. These significant differences in resistance profiles among species emphasize the need for species-specific antifungal treatment strategies.

Concern over tolerance toward FLC is significant (Table 2 and Table 3), as FLC is a frequently prescribed azole for addressing disseminated candidiasis, including bloodstream infection (candidemia), as an oral formulation. The widespread use of FLC in diverse medical settings is the primary reason for its superiority against *C. albicans* [76]. Despite increasing azole resistance, voriconazole retained >90% susceptibility in our isolates (Table 4), consistent with European surveillance data reporting preserved activity of second-generation azoles [77]. Additionally, except for the three *C. albicans* isolates that demonstrated resistance, all remaining isolates across species were susceptible to this peculiar antifungal. Importantly, voriconazole also remains a valuable option against *C. glabrata*, a species that has globally exhibited high rates of FLC resistance [17]. Given this consistently high susceptibility, voriconazole represents a rational and evidence-supported step-down therapy, particularly in settings where FLC resistance is of concern.

More than 90% itraconazole resistance was detected in *C. glabrata*, consistent with its capacity to rapidly develop azole resistance through overexpression of ATP-binding cassette (ABC) and major facilitator superfamily (MFS) efflux pumps (e.g., CDR1, CDR2) [78]. These mechanisms frequently confer cross-resistance across triazoles, which was evident in our study where *C. glabrata* showed multi-azole-resistant towards ketoconazole, fluconazole, itraconazole, and posaconazole (Table 5), in line with global resistance trends. National Chinese surveillance further reports 1.6% cross-resistance to fluconazole and voriconazole, driven by efflux-pump activity and *ERG11* (lanosterol 14-α-demethylase) alterations [79]. Severe multi-drug-resistant (MDR) phenotypes have also been documented, including cases where all bloodstream isolates were resistant to fluconazole, voriconazole, and all echinocandins, indicating emerging pan-resistant strains [80]. Additional in vitro data similarly report significant triple-azole resistance [81]. These patterns are more common in patients with prolonged azole prophylaxis. Collectively, these findings underscore the need for caution when using itraconazole or other azoles for *C. glabrata* infections and highlight the importance of ongoing local susceptibility surveillance to limit further selection of highly resistant strains.

Multi-azole-resistant strains of *C. parapsilosis* (Table 5) has emerged as a significant clinical concern, driven by *ERG11* mutations, *FKS1* (1,3-β-D-glucan synthase catalytic subunit) alterations, and efflux-pump overexpression (*MDR1*, *CDR1*), leading to FLC resistance; thus, contributing to persistent hospital outbreaks [82]. Similarly, pan-azole–resistant *C. albicans* has been reported in rare clinical cases, usually associated with combined *ERG11/ERG3* mutations and upregulation of *CDR/MDR* efflux pumps [83]. Although such highly resistant *C. albicans* strains can arise through these molecular mechanisms, they remain uncommon in large surveillance datasets, underscoring the importance of continued molecular monitoring and routine antifungal susceptibility testing.

In the rarest case, one patient was coinfected with two *Candida* species (Table 6). Although exceptionally uncommon, a previous study has reported concomitant isolation of *C. albicans* and *C. glabrata* from patients with oral candidiasis [84]. Moreover, the simultaneous presence of both *C. albicans* and *C. glabrata* is associated with increased pathogenicity [85]. Therefore, the dual-resistance pattern exhibited by these coinfecting species raises serious concerns for patients and warrants careful monitoring and thorough management of candidemia.

In our study, both AmB and caspofungin showed 100% activity against NAC isolates (Table 3 and Table 4), consistent with Global ARTEMIS and SENTRY surveillance data [47,79,86], although AmB’s nephrotoxicity limits its first-line use. Current guidelines advocate the use of caspofungin as the initial therapy for candidemia in both neutropenic and non-neutropenic patients—and as preferred empiric therapy in critically ill ICU patients, owing to their safety profile, fungicidal activity, and rising azole resistance. Step-down to FLC within 5–7 days is advised only when patients are clinically stable, blood cultures are negative, and the infecting species demonstrates FLC susceptibility. For species exhibiting azole resistance or for NAC isolates with demonstrated susceptibility, voriconazole may be considered as a step-down or alternative therapy. Since *C. glabrata* is strongly associated with prior FLC exposure, invasive systemic infections require prompt initiation of echinocandin therapy without early de-escalation. The emergence of azole-resistant *C. glabrata* and *C. parapsilosis* in our study reinforces the need for susceptibility-guided therapy and aligns with the IDSA 2016 emphasis [87] and the Middle East regional guidelines [24] on early echinocandin use. Further, AmB remains reserved for resistant, refractory, or breakthrough infections, especially when azole or echinocandin resistance is suspected or confirmed.

### Limitations

This study poses several limitations. Risk-factor data for patients were incomplete, and the sample size was too small to exclude potential bias. Documentation variability limited access to certain clinical details (such as infection site, sepsis status and comorbidities), although essential data required for the study’s objectives remained available. MIC results were obtained without correlation to clinical course or antifungal therapy, preventing assessment of the impact of FLC resistance on patient outcomes. Moreover, the absence of susceptibility testing for one or more antifungals in some isolates further limits the generalizability of the findings. Additionally, genomic sequencing of resistant isolates was not performed, restricting insights into underlying resistance mechanisms. Larger studies with comprehensive clinical data and integrated molecular analyses are needed to confirm and expand these findings.

## 5. Conclusions

Identifying fungal pathogens in clinical samples and determining their antifungal resistance patterns are essential for optimizing clinical outcomes and selecting suitable and effective antifungal therapies. In the present study, *C. glabrata* and *C. parapsilosis* emerged as the predominant isolates in the NAC group, with *C. glabrata* showing the highest resistance. Furthermore, the observed resistance of *C. albicans* to all tested antifungals raises significant concerns regarding the effective management of nosocomial bloodstream infections. Moreover, our study concluded that voriconazole may be a better option than FLC for treating disseminated candidiasis including bloodstream candidemia. Healthcare professionals can use this information to suggest reliable treatment options for *Candida* bloodstream infections caused by NAC. An unusual case of coinfection involving both *C. glabrata* and *C. albicans* displayed a dual-resistance pattern against posaconazole and itraconazole. Clinicians may need to consider alternative therapeutic strategies, closely monitor patient responses, and reassess selected antifungal regimens based on identified resistance patterns.

## Figures and Tables

**Table 1:** Demographic data of patients diagnosed with fungal infection.

Characteristics		Number (%)
Sex	Males	28 (50.9%)
	Females	27 (49.1%)
Age	<2	7 (12.8%)
	2–6	0 (0.0%)
	7–12	6 (10.9%)
	13–19	3 (5.4%)
	20–64	26 (47.2%)
	>65	13 (23.7%)
Infection	*C. albicans*	11 (20.0%)
	*C. glabrata*	11 (20.0%)
	*C. parapsilosis*	10 (18.2%)
	*C. tropicalis*	6 (10.9%)
	*C. haemulonii*	5 (9.1%)
	*C. auris*	3 (5.4%)
	*C. famata*	1 (1.8%)
	*C. rugosa*	1 (1.8%)
	*C. lustinae*	1 (1.8%)
	*Rhodotorula glutins*	3 (5.4%)
	*Trichosposron*	2 (3.64)
	*C. glabrata* and *C. albicans* Co-infection	1 (1.8%)
Samples collection	Aerobic vial	30 (54.5%)
	Anaerobic vial	4 (7.3%)
	Both aerobic and anaerobic vial	15 (27.2%)
	Pediatric vial	6 (10.9%)
Site of collection	ICU	15 (27.3%)
	Hospital wards	40 (72.7%)

**Table 2:** Antifungal profiling in males and females.

Antifungal (n *)	Males			Females		
	Susceptible (%)	Resistant (%)	Intermediate (%)	Susceptible (%)	Resistant (%)	Intermediate (%)
**Fluconazole (55)**	10 (18.2)	17 ^#^ (30.1)	1 (1.8)	8 (1.4)	17 ^#^ (30.1)	2 (3.6)
**Itraconazole (54)**	9 (16.7)	6 (11.1)	1 (1.85)	21 (38.9)	6 (11.1)	0 (0.0)
**Posaconazole (28)**	9 (32.1)	4 (14.2)	0 (0.0)	14 (50.0)	1 (3.5)	0 (0.0)
**Ketoconazole (55)**	26 (47.3)	2 (3.6)	0 (0.0)	24 (43.6)	3 (5.4)	0 (0.0)
**Voriconazole (28)**	9 (32.1)	3 (10.7)	0 (0.0)	14 (50.0)	0 (0.0)	2 (7.1)
**Amphotericin B (54)**	27 (50.0)	0 (0.0)	0 (0.0)	27 (50.0)	0 (0.0)	0 (0.0)
**Caspofungin (3)**	2 (66.7)	0 (0.0)	0 (0.0)	1 (33.3)	0 (0.0)	0 (0.0)

* Number of samples tested for that particular antifungal. ^#^ *p* value < 0.05.

**Table 3:** Antifungal susceptibility against fungal pathogens (n = 54) *.

Anti-Fungal	*C.* *albicans* (11) ^a^			*C. auris* (3)			*C.* *glabrata* (11)			*C.* *tropicalis* (6)			*C. parapsilosis* (10)			*C.* *haemulonii* (5)			*C. famata* (1)			*C. rugosa* (1)			*C. lustinae* (1)			*Trichosposron* (2)			*R. glutins* (3)		
	S	I	R	S	I	R	S	I	R	S	I	R	S	I	R	S	I	R	S	I	R	S	I	R	S	I	R	S	I	R	S	I	R
**P**	5	0	1	1	0	0	0	0	4	4	0	0	4	0	0	1	0	0	1	0	0	1	0	0	1	0	0	2	0	0	3	0	0
**K**	10	0	1	3	0	0	8	0	3	6	0	0	9	0	1	5	0	0	1	0	0	1	0	0	1	0	0	2	0	0	3	0	0
**V**	4	0	3	1	0	0	4	0	0	2	1	0	3	1	0	1	0	0	1	0	0	1	0	0	1	0	0	2	0	0	3	0	0
**F**	5	1	5	0	0	3	0	1	10	4	0	2	3	1	6	0	0	5	1	0	0	1	0	0	1	0	0	2	0	0	0	0	3
**I**	10	0	1	3	0	0	1	1	9	5	0	1	9	0	1	5	0	0	1	0	0	1	0	0	n	0	0	2	0	0	3	0	0
**AmB**	11	0	0	3	0	0	1	0	0	6	0	0	0	0	0	5	0	0	1	0	0	1	0	0	1	0	0	2	0	0	3	0	0
**Cas**	1	0	0	n	n	n	2	0	0	n	n	n	n	n	n	n	n	n	n	n	n	n	n	n	n	n	n	n	n	n	n	n	n

P, Posaconazole; K, Ketoconazole; V, Voriconazole; F, Fluconazole; I, Itraconazole; AmB, Amphotericin B; Cas, Caspofungin. S, susceptible; I, intermediate; R, resistant; n, not determined. ^a^ Values in brackets indicate the total number of isolates. * The data exclude the patient co-infected with *C. albicans* and *C. glabrata*. Note: Not all isolates of each species were tested against every antifungal agent.

**Table 4:** In vitro activities of antifungal agents (mg/mL) tested against fungal species (55 isolates).

Species (n) ^a^	MIC Range/Value (mg/L)						
	PCZ	KCZ	VRC	ITZ	FLC	AmB	CAS
***C. albicans*** (11/1 *)	0.0625–32	0.0312–32	0.0156–16	0.0312–32	0.125–64	0.125–0.5	0.125–0.5
***C. glabrata*** (11/1 *)	32–64	0.0312–32	0.125–0.5	16–32	16–64	0.125–0.5	0.0625–0.125
***C. parapsilosis*** **(10)**	0.25–0.5	0.0312–32	0.0312–0.125	0.25–32	0.0625–32	0.125–0.5	ND ^1^
***C. tropicalis*** **(6)**	0.0312–0.25	0.0625–0.25	0.0625–0.25	0.125–32	0.25–64	0.125–0.5	ND
***C. haemulonii*** **(5)**	0.0312–0.125	0.0625–0.125	0.0312–0.25	0.0625–0.5	0.5–32	0.125–0.5	ND
***C. auris*** **(3)**	0.0312–0.5	0.0156–0.5	0.0156–0.25	0.0312–0.25	16–64	0.125–0.25	ND
***C. rugosa*** **(1)**	0.25	0.0312	0.0625	0.5	0.25	0.25	ND
***C. lustinae*** **(1)**	0.0625	0.0312	0.0312	ND	0.25	0.125	ND
***C. famata*** **(1)**	0.0625	0.0312	0.125	0.25	0.25	0.0625	ND
***R. glutins*** **(3)**	0.0625–0.5	0.125–0.0625	0.125	0.125–0.25	32	0.125	ND
***Trichosposron* (2)**	0.125–0.5	0.125	0.0625–0.125	0.5	0.125–0.25	0.125	ND

PCZ, Posaconazole; KCZ, Ketoconazole; VRC, Voriconazole; ITZ, Itraconazole; FLC, Fluconazole; AmB, Amphotericin B; CAS, Caspofungin; ^a^ Values in brackets indicate the total number of isolates; * Co-infection; ^1^ ND, not determined.

**Table 5:** Correlation of multi-azole resistant pattern with *Candida* species.

Resistance Pattern	Species (n) ^a^
**Double resistant (7)**	
Posaconazole/Fluconazole	*C. glabrata* (1)
Posaconazole/Itraconazole	*C. albicans* and *C. glabrata* co-infection (1)
Voriconazole/Fluconazole	*C. albicans* (2)
Itraconazole/Fluconazole	*C. glabrata* (2), *C. tropicalis* (1)
**Triple resistant (7)**	
Ketoconazole/Fluconazole/Itraconazole	*C. glabrata* (3), *C. parapsilosis* (1)
Posaconazole/Fluconazole/Itraconazole	*C. glabrata* (3)
**Penta-resistant (1)**	
Posaconazole/Ketoconazole/fluconazole/Itraconazole/voriconazole	*C. albicans* (1)

^a^ Values in parenthesis indicate the total number of isolates.

**Table 6:** Antifungal susceptibility pattern in a patient co-infected with *C. albicans* and *C. glabrata*.

Species	Posaconazole	Ketoconazole	Voriconazole	Fluconazole	Itraconazole	Amphotericin B	Caspofungin
*C. albicans*	**R (32) ^a^**	S	S	S	**R (32)**	S	ND ^1^
*C. glabrata*	**R (32)**	S	S	I	**R (32)**	S	ND

**^a^** The value is parenthesis represents the MIC for the particular antifungal; ^1^ ND, not determined.

## Data Availability

The original contributions presented in this study are included in the article/Supplementary Material. Further inquiries can be directed to the author.

## Supplementary Materials

The following supporting information can be downloaded at: https://www.mdpi.com/article/10.3390/pathogens14121221/s1, Figure S1: Antifungal resistance pattern of clinical fungal isolates; Table S1: Infection profiling in males and females.

## Abbreviations

The following abbreviations are used in this manuscript:

FIs	Fungal infections
ICUs	Intensive care units
KSA	Kingdon of Saudi Arabia
NAC	non-albicans *Candida*
AmB	Amphotericin B
CLSI	Clinical and Laboratory Standards Institute
FLC	Fluconazole
Cas	Caspofungin
ECDC	European Centre for Disease Prevention and Control
ABC	ATP-binding cassette
MFS	Major facilitator superfamily
MDR	Multi-drug resistant

## References

[1] Rokas A. (2022). Evolution of the Human Pathogenic Lifestyle in Fungi. Nat. Microbiol..

[2] Gnat S., Łagowski D., Nowakiewicz A., Dyląg M. (2021). A Global View on Fungal Infections in Humans and Animals: Infections Caused by Dimorphic Fungi and Dermatophytoses. J. Appl. Microbiol..

[3] Bongomin F., Gago S., Oladele R.O., Denning D.W. (2017). Global and Multi-National Prevalence of Fungal Diseases—Estimate Precision. J. Fungi.

[4] Kainz K., Bauer M.A., Madeo F., Carmona-Gutierrez D. (2020). Fungal Infections in Humans: The Silent Crisis. Microb. Cell.

[5] Al Dhaheri F., Thomsen J., Everett D., Denning D.W. (2024). Mapping the Burden of Fungal Diseases in the United Arab Emirates. J. Fungi.

[6] Kmeid J., Jabbour J.F., Kanj S.S. (2020). Epidemiology and Burden of Invasive Fungal Infections in the Countries of the Arab League. J. Infect. Public Health.

[7] Alkhalifa W., Alnimr A., Alhawaj H., Alamri A., Alturki F., Alshahrani M. (2023). Clinical and Microbiological Characteristics of Candidemia Cases in Saudi Arabia. Infect. Drug Resist..

[8] Taj-Aldeen S.J., Chandra P., Denning D.W. (2015). Burden of Fungal Infections in Qatar. Mycoses.

[9] Kazak E., Akın H., Ener B., Sığırlı D., Özkan O., Gürcüoğlu E., Yılmaz E., Çelebi S., Akçağlar S., Akalın H. (2014). An Investigation of *Candida* Species Isolated from Blood Cultures during 17 Years in a University Hospital. Mycoses.

[10] Sutcu M., Salman N., Akturk H., Dalgıc N., Turel O., Kuzdan C., Kadayifci E.K., Sener D., Karbuz A., Erturan Z. (2016). Epidemiologic and Microbiologic Evaluation of Nosocomial Infections Associated with *Candida* spp in Children: A Multicenter Study from Istanbul, Turkey. Am. J. Infect. Control.

[11] Yeşilkaya A., Azap Ö., Aydın M., Akçil Ok M. (2017). Epidemiology, Species Distribution, Clinical Characteristics and Mortality of Candidaemia in a Tertiary Care University Hospital in Turkey, 2007–2014. Mycoses.

[12] Alobaid K., Khan Z. (2019). Epidemiologic Characteristics of Adult Candidemic Patients in a Secondary Hospital in Kuwait: A Retrospective Study. J. Mycol. Med..

[13] Osman M., Al Bikai A., Rafei R., Mallat H., Dabboussi F., Hamze M. (2020). Update on Invasive Fungal Infections in the Middle Eastern and North African Region. Braz. J. Microbiol..

[14] Omrani A.S., Makkawy E.A., Baig K., Baredhwan A.A., Almuthree S.A., Elkhizzi N.A., Albarrak A.M. (2014). Ten-Year Review of Invasive *Candida* Infections in a Tertiary Care Center in Saudi Arabia. Saudi Med. J..

[15] Ala-Houhala M., Valkonen M., Kolho E., Friberg N., Anttila V.J. (2019). Clinical and Microbiological Factors Associated with Mortality in Candidemia in Adult Patients 2007–2016. Infect. Dis..

[16] Kullberg B.J., Arendrup M.C. (2015). Invasive Candidiasis. N. Engl. J. Med..

[17] Costa-de-oliveira S., Rodrigues A.G. (2020). *Candida albicans* Antifungal Resistance and Tolerance in Bloodstream Infections: The Triad Yeast-Host-Antifungal. Microorganisms.

[18] Papon N., Courdavault V., Clastre M., Bennett R.J. (2013). Emerging and Emerged Pathogenic *Candida* Species: Beyond the *Candida albicans* Paradigm. PLoS Pathog..

[19] Megri Y., Arastehfar A., Boekhout T., Daneshnia F., Hörtnagl C., Sartori B., Hafez A., Pan W., Lass-Flörl C., Hamrioui B. (2020). *Candida tropicalis* Is the Most Prevalent Yeast Species Causing Candidemia in Algeria: The Urgent Need for Antifungal Stewardship and Infection Control Measures. Antimicrob. Resist. Infect. Control..

[20] Lamoth F., Lockhart S.R., Berkow E.L., Calandra T. (2018). Changes in the Epidemiological Landscape of Invasive Candidiasis. J. Antimicrob. Chemother..

[21] Gómez-Gaviria M., Ramírez-Sotelo U., Mora-Montes H.M. (2022). Non-Albicans *Candida* Species: Immune Response, Evasion Mechanisms, and New Plant-Derived Alternative Therapies. J. Fungi.

[22] Parslow B.Y., Thornton C.R. (2022). Continuing Shifts in Epidemiology and Antifungal Susceptibility Highlight the Need for Improved Disease Management of Invasive Candidiasis. Microorganisms.

[23] Husni R., Bou Zerdan M., Samaha N., Helou M., Mahfouz Y., Saniour R., Hourani S., Kolanjian H., Afif C., Azar E. (2023). Characterization and Susceptibility of Non-Albicans *Candida* Isolated from Various Clinical Specimens in Lebanese Hospitals. Front. Public Health.

[24] Alothman A.F., Al-Musawi T., Al-Abdely H.M., Al Salman J., Almaslamani M., Yared N., Butt A.A., Raghubir N., El Morsi W., Al Thaqafi A.O. (2014). Clinical Practice Guidelines for the Management of Invasive *Candida* Infections in Adults in the Middle East Region: Expert Panel Recommendations. J. Infect. Public Health.

[25] Khateb A.M., Alofi F.S., Almutairi A.Z. (2023). Increased Prevalence of Fungemia in Medina, Saudi Arabia. Front. Epidemiol..

[26] Khateb A.M., Alofi F.S., Alturkostani M.A., Almutairi A.Z. (2025). Shifting Sands: Unveiling the Changes in Respiratory Comorbidities and Fungal Pathogens in Saudi Arabia. Saudi Med. J..

[27] Alkharashi N., Aljohani S., Layqah L., Masuadi E., Baharoon W., Al-Jahdali H., Baharoon S. (2019). *Candida* Bloodstream Infection: Changing Pattern of Occurrence and Antifungal Susceptibility over 10 Years in a Tertiary Care Saudi Hospital. Can. J. Infect. Dis. Med. Microbiol..

[28] Almoosa Z., Ahmed G.Y., Omran A., Alsarheed A., Alturki A., Alaqeel A., Alshehri M., Alfawaz T., Alshahrani D. (2017). Invasive Candidiasis in Pediatric Patients at King Fahad Medical City in Central Saudi Arabia. A 5-Year Retrospective Study. Saudi Med. J..

[29] Mota Fernandes C., Dasilva D., Haranahalli K., McCarthy J.B., Mallamo J., Ojima I., Del Poeta M. (2020). The Future of Antifungal Drug Therapy: Novel Compounds and Targets. Antimicrob. Agents Chemother..

[30] Anderson T.M., Clay M.C., Cioffi A.G., Diaz K.A., Hisao G.S., Tuttle M.D., Nieuwkoop A.J., Comellas G., Maryum N., Wang S. (2014). Amphotericin Forms an Extramembranous and Fungicidal Sterol Sponge. Nat. Chem. Biol..

[31] Houšť J., Spížek J., Havlíček V. (2020). Antifungal Drugs. Metabolites.

[32] Hasim S., Coleman J.J. (2019). Targeting the Fungal Cell Wall: Current Therapies and Implications for Development of Alternative Antifungal Agents. Futur. Med. Chem..

[33] Ding X., Yan D., Sun W., Zeng Z., Su R., Su J. (2015). Epidemiology and Risk Factors for Nosocomial Non-*Candida albicans* Candidemia in Adult Patients at a Tertiary Care Hospital in North China. Med. Mycol..

[34] Al-Baqsami Z.F., Ahmad S., Khan Z. (2020). Antifungal Drug Susceptibility, Molecular Basis of Resistance to Echinocandins and Molecular Epidemiology of Fluconazole Resistance among Clinical *Candida glabrata* Isolates in Kuwait. Sci. Rep..

[35] Doern G.V., Carroll K.C., Diekema D.J., Garey K.W., Rupp M.E., Weinstein M.P., Sextong D.J. (2019). Practical Guidance for Clinical Microbiology Laboratories: A Comprehensive Update on the Problem of Blood Culture Contamination and a Discussion of Methods for Addressing the Problem. Clin. Microbiol. Rev..

[36] CLSI (2022). M47-Principles and Procedures for Blood Cultures Suggested Citation.

[37] Graf B., Adam T., Zill E., Göbel U.B. (2000). Evaluation of the VITEK 2 System for Rapid Identification of Yeasts and Yeast-like Organisms. J. Clin. Microbiol..

[38] (2017). Reference Method for Broth Dilution Antifungal Susceptibility Testing of Yeasts.

[39] (2022). Performance Standards for Antifungal Susceptibility Testing of Yeasts.

[40] Al-Hedaithy S.S.A. (2003). The Yeast Species Causing Fungemia at a University Hospital in Riyadh, Saudi Arabia, during a 10-Year Period. Mycoses.

[41] Akbar D.H., Tahawi A.T. (2001). Candidemia at a University Hospital: Epidemiology, Risk Factors and Predictors of Mortality. Ann. Saudi Med..

[42] Al-Musawi T.S., Alkhalifa W.A., Alasaker N.A., Rahman J.U., Alnimr A.M. (2021). A Seven-Year Surveillance of *Candida* Bloodstream Infection at a University Hospital in KSA. J. Taibah Univ. Med. Sci..

[43] Marchetti O., Bille J., Fluckiger U., Eggimann P., Ruef C., Garbino J., Calandra T., Glauser M.P., Täuber M.G., Pittet D. (2004). Epidemiology of Candidemia in Swiss Tertiary Care Hospitals: Secular Trends, 1991–2000. Clin. Infect. Dis..

[44] Sipsas N.V., Lewis R.E., Tarrand J., Hachem R., Rolston K.V., Raad I.I., Kontoyiannis D.P. (2009). Candidemia in Patients with Hematologic Malignancies in the Era of New Antifungal Agents (2001–2007): Stable Incidence but Changing Epidemiology of a Still Frequently Lethal Infection. Cancer.

[45] Pfaller M.A., Diekema D.J. (2010). Epidemiology of Invasive Mycoses in North America. Crit. Rev. Microbiol..

[46] Pfaller M.A., Diekema D.J., Jones R.N., Sader H.S., Fluit A.C., Hollis R.J., Messer S.A. (2001). International Surveillance of Bloodstream Infections Due to *Candida* Species: Frequency of Occurrence and in Vitro Susceptibilities to Fluconazole, Ravuconazole, and Voriconazole of Isolates Collected from 1997 through 1999 in the SENTRY Antimicrobial Surveillance Program. J. Clin. Microbiol..

[47] Pfaller M.A., Diekema D.J., Turnidge J.D., Castanheira M., Jones R.N. (2019). Twenty Years of the SENTRY Antifungal Surveillance Program: Results for *Candida* Species From 1997–2016. Open Forum Infect. Dis..

[48] Pfaller M.A., Carvalhaes C.G., DeVries S., Huband M.D., Castanheira M. (2022). Elderly versus Nonelderly Patients with Invasive Fungal Infections: Species Distribution and Antifungal Resistance, SENTRY Antifungal Surveillance Program 2017–2019. Diagn. Microbiol. Infect. Dis..

[49] Pfaller M.A., Moet G.J., Messer S.A., Jones R.N., Castanheira M. (2011). *Candida* Bloodstream Infections: Comparison of Species Distributions and Antifungal Resistance Patterns in Community-Onset and Nosocomial Isolates in the SENTRY Antimicrobial Surveillance Program, 2008–2009. Antimicrob. Agents Chemother..

[50] Biswas C., Marcelino V.R., Van Hal S., Halliday C., Martinez E., Wang Q., Kidd S., Kennedy K., Marriott D., Morrissey C.O. (2018). Whole Genome Sequencing of Australian *Candida glabrata* Isolates Reveals Genetic Diversity and Novel Sequence Types. Front. Microbiol..

[51] Koehler P., Stecher M., Cornely O.A., Koehler D., Vehreschild M.J.G.T., Bohlius J., Wisplinghoff H., Vehreschild J.J. (2019). Morbidity and Mortality of Candidaemia in Europe: An Epidemiologic Meta-Analysis. Clin. Microbiol. Infect..

[52] Tan T.Y., Hsu L.Y., Alejandria M.M., Chaiwarith R., Chinniah T., Chayakulkeeree M., Choudhury S., Chen Y.H., Shin J.H., Kiratisin P. (2016). Antifungal Susceptibility of Invasive *Candida* Bloodstream Isolates from the Asia-Pacific Region. Med. Mycol..

[53] Tan B.H., Chakrabarti A., Li R.Y., Patel A.K., Watcharananan S.P., Liu Z., Chindamporn A., Tan A.L., Sun P.L., Wu U.I. (2015). Incidence and Species Distribution of Candidaemia in Asia: A Laboratory-Based Surveillance Study. Clin. Microbiol. Infect..

[54] Chen Y.C., Dhillon S., Adomakoh N., Roberts J.A. (2025). The Changing Epidemiology of *Candida* Species in Asia Pacific and Evidence for Optimizing Antifungal Dosing in Challenging Clinical Scenarios. Expert. Rev. Anti-Infect. Ther..

[55] Meng L., Li J., Wang D., Han M., Gao S., Zhang Y., Zhu W., Liu C. (2025). Epidemiology, Risk Factors, and Antifungal Susceptibility Analysis of *Candida tropicalis* and Non-*C. tropicalis* Candidemia. BMC Infect. Dis..

[56] Ann Chai L.Y., Denning D.W., Warn P. (2010). *Candida tropicalis* in Human Disease. Crit. Rev. Microbiol..

[57] Yesudhason B.L., Mohanram K. (2015). *Candida tropicalis* as a predominant isolate from clinical specimens and its antifungal susceptibility pattern in a tertiary care hospital in southern India. J. Clin. Diagn. Res..

[58] Kwon Y.J., Won E.J., Jeong S.H., Shin K.S., Shin J.H., Kim Y.R., Kim H.S., Kim Y.A., Uh Y., Kim T.S. (2021). Dynamics and Predictors of Mortality Due to Candidemia Caused by Different *Candida* Species: Comparison of Intensive Care Unit-Associated Candidemia (Icuac) and Non-Icuac. J. Fungi.

[59] Aldardeer N.F., Albar H., Al-Attas M., Eldali A., Qutub M., Hassanien A., Alraddadi B. (2020). Antifungal Resistance in Patients with Candidaemia: A Retrospective Cohort Study. BMC Infect. Dis..

[60] Bukharie H.A. (2002). Nosocomial Candidemia in a Tertiary Care Hospital in Saudi Arabia. Mycopathologia.

[61] Won E.J., Shin J.H., Choi M.J., Lee W.G., Park Y.J., Uh Y., Kim S.Y., Lee M.K., Kim S.H., Shin M.G. (2015). Antifungal Susceptibilities of Bloodstream Isolates of *Candida* Species from Nine Hospitals in Korea: Application of New Antifungal Breakpoints and Relationship to Antifungal Usage. PLoS ONE.

[62] Ramos L.S., Figueiredo-Carvalho M.H.G., Silva L.N., Siqueira N.L.M., Lima J.C., Oliveira S.S., Almeida-Paes R., Zancopé-Oliveira R.M., Azevedo F.S., Ferreira A.L.P. (2022). The Threat Called *Candida haemulonii* Species Complex in Rio de Janeiro State, Brazil: Focus on Antifungal Resistance and Virulence Attributes. J. Fungi.

[63] Beyda N.D., Chuang S.H., Jahangir Alam M., Shah D.N., Ng T.M., McCaskey L., Garey K.W. (2013). Treatment of *Candida famata* Bloodstream Infections: Case Series and Review of the Literature. J. Antimicrob. Chemother..

[64] Ludwig A., de Jesus F.P.K., Dutra V., Cândido S.L., Alves S.H., Santurio J.M. (2019). Susceptibility Profile of *Candida rugosa* (*Diutina rugosa*) against Antifungals and Compounds of Essential Oils. J. Mycol. Med..

[65] Khan Z., Ahmad S., Al-Sweih N., Khan S., Joseph L. (2019). *Candida lusitaniae* in Kuwait: Prevalence, Antifungal Susceptibility and Role in Neonatal Fungemia. PLoS ONE.

[66] Khan Z.U., Al-Sweih N.A., Ahmad S., Al-Kazemi N., Khan S., Joseph L., Chandy R. (2007). Outbreak of Fungemia among Neonates Caused by *Candida haemulonii* Resistant to Amphotericin B, Itraconazole, and Fluconazole. J. Clin. Microbiol..

[67] Alshahrani F.S., Elgujja A.A., Alsubaie S., Ezreqat S.A., Albarraq A.M., Barry M., Binkhamis K., Alabdan L. (2023). Description of *Candida auris* Occurrence in a Tertiary Health Institution in Riyadh, Saudi Arabia. Healthcare.

[68] Munshi A., Almadani F., Ossenkopp J., Alharbi M., Althaqafi A., Alsaedi A., Al-Amri A., Almarhabi H. (2024). Risk Factors, Antifungal Susceptibility, Complications, and Outcome of *Candida auris* Bloodstream Infection in a Tertiary Care Center in the Western Region of Saudi Arabia. J. Infect. Public Health.

[69] European Centre for Disease Prevention and Control (2025). Survey on the Epidemiological Situation, Laboratory Capacity and Preparedness for Candidozyma (Candida) Auris, 2024.

[70] Hato H., Sakata K.I., Sato J., Hasebe A., Yamazaki Y., Kitagawa Y. (2022). Factor Associated with Oral Candidiasis Caused by Co-Infection of *Candida albicans* and *Candida glabrata*: A Retrospective Study. J. Dent. Sci..

[71] McHugh J., Chesdachai S., Dunsirn M., Wengenack N., Vergidis P. (2025). Increasing Fluconazole Resistance in *Candida parapsilosis*: A 10-Year Analysis of Blood Culture Isolates at a US Reference Laboratory (2015–2024). J. Infect. Dis..

[72] Lee Y., Robbins N., Cowen L.E. (2023). Molecular Mechanisms Governing Antifungal Drug Resistance. npj Antimicrob. Resist..

[73] Kumar A., Prakash A., Singh A., Kumar H., Hagen F., Meis J.F., Chowdhary A. (2016). *Candida haemulonii* Species Complex: An Emerging Species in India and Its Genetic Diversity Assessed with Multilocus Sequence and Amplified Fragment-Length Polymorphism Analyses. Emerg. Microbes Infect..

[74] Spiliopoulou A., Anastassiou E.D., Christofidou M. (2012). Rhodotorula Fungemia of an Intensive Care Unit Patient and Review of Published Cases. Mycopathologia.

[75] Miglietta F., Faneschi M.L., Braione A., Palumbo C., Rizzo A., Lobreglio G., Pizzolante M. (2015). Central Venous Catheter-Related Fungemia Caused by Rhodotorula Glutinis. Med. Mycol. J..

[76] Delarze E., Brandt L., Trachsel E., Patxot M., Pralong C., Maranzano F., Chauvel M., Legrand M., Znaidi S., Bougnoux M.E. (2020). Identification and Characterization of Mediators of Fluconazole Tolerance in *Candida albicans*. Front. Microbiol..

[77] Odoj K., Garlasco J., Pezzani M.D., Magnabosco C., Ortiz D., Manco F., Galia L., Foster S.K., Arieti F., Tacconelli E. (2024). Tracking Candidemia Trends and Antifungal Resistance Patterns across Europe: An In-Depth Analysis of Surveillance Systems and Surveillance Studies. J. Fungi.

[78] Safdar A., Chaturvedi V., Koll B.S., Larone D.H., Perlin D.S., Armstrong D. (2002). Prospective, Multicenter Surveillance Study of *Candida glabrata*: Fluconazole and Itraconazole Susceptibility Profiles in Bloodstream, Invasive, and Colonizing Strains and Differences between Isolates from Three Urban Teaching Hospitals in New York City (*Candida* Susceptibility Trends Study, 1998 to 1999). Antimicrob. Agents Chemother..

[79] Xiao M., Fan X., Chen S.C.A., Wang H., Sun Z.Y., Liao K., Chen S.L., Yan Y., Kang M., Hu Z.D. (2015). Antifungal Susceptibilities of *Candida glabrata* Species Complex, *Candida krusei*, *Candida parapsilosis* Species Complex and *Candida tropicalis* Causing Invasive Candidiasis in China: 3 Year National Surveillance. J. Antimicrob. Chemother..

[80] Thompson G.R., Wiederhold N.P., Vallor A.C., Villareal N.C., Lewis J.S., Patterson T.F. (2008). Development of Caspofungin Resistance Following Prolonged Therapy for Invasive Candidiasis Secondary to *Candida glabrata* Infection. Antimicrob. Agents Chemother..

[81] Amirrajab N., Badali H., Didehdar M., Afsarian M.H., Mohammadi R., Lotfi N., Shokohi T., Amirrajab N., Badali H., Didehdar M. (2016). In Vitro Activities of Six Antifungal Drugs Against *Candida glabrata* Isolates: An Emerging Pathogen. Jundishapur J. Microbiol..

[82] Daneshnia F., Floyd D.J., Ryan A.P., Ghahfarokhy P.M., Ebadati A., Jusuf S., Munoz J., Jeffries N.E., Elizabeth Yvanovich E., Apostolopoulou A. (2024). Evaluation of Outbreak Persistence Caused by Multidrug-Resistant and Echinocandin-Resistant *Candida parapsilosis* Using Multidimensional Experimental and Epidemiological Approaches. Emerg. Microbes Infect..

[83] Keighley C., Gall M., Halliday C.L., Chaw K., Newton P., Sintchenko V., Chen S.C.A. (2024). Breakthrough *Candida albicans* Bloodstream Infection Associated with in Vivo Development of Pan-Azole Resistance Related to *ERG3* Gene Deletion. Pathology.

[84] Okada K., Nakazawa S., Yokoyama A., Kashiwazaki H., Kobayashi K., Yamazaki Y. (2016). Clinical Study of Mixed Infection with *Candida albicans* and *Candida glabrata* in Oral Candidiasis. Geriatr. Dent..

[85] Coco B.J., Bagg J., Cross L.J., Jose A., Cross J., Ramage G. (2008). Mixed *Candida albicans* and *Candida glabrata* Populations Associated with the Pathogenesis of Denture Stomatitis. Oral. Microbiol. Immunol..

[86] Pfaller M.A., Messer S.A., Woosley L.N., Jones R.N., Castanheira M. (2013). Echinocandin and Triazole Antifungal Susceptibility Profiles for Clinical Opportunistic Yeast and Mold Isolates Collected from 2010 to 2011: Application of New CLSI Clinical Breakpoints and Epidemiological Cutoff Values for Characterization of Geographic and temporal Trends of Antifungal Resistance. J. Clin. Microbiol..

[87] Pappas P.G., Kauffman C.A., Andes D.R., Clancy C.J., Marr K.A., Ostrosky-Zeichner L., Reboli A.C., Schuster M.G., Vazquez J.A., Walsh T.J. (2016). Clinical Practice Guideline for the Management of Candidiasis: 2016 Update by the Infectious Diseases Society of America. Clin. Infect. Dis..

