# Supplementary material for: Epidemiology of Fungal Bloodstream Infections and Antifungal Susceptibility in a Tertiary Care Hospital in Riyadh, Saudi Arabia: A Rare Candida Co-Infection Case
Source: Pathogens. 2025 Nov 30;14(12):1221. doi: 10.3390/pathogens14121221 (PMC12736276; doi:10.3390/pathogens14121221)
Supplement: Supplementary file 1 [file pathogens-14-01221-s001.zip › pathogens-3940411-supplementary.pdf]

Supplementary data

Table S1. Infection profiling in males and females

Candida **spp.**	**Males (n)**	**Females (n)**
C. lustinae	1	0
C. rugosa	0	1
C. famata	1	0
C. auris	1	2
C. haemulonii	4	1
C. albicans	6	5
C. parapsilosis	8	2
C. grabrata	5	6
C. tropicalis	1	5
Trichosposron	0	2
R. glutins	0	3

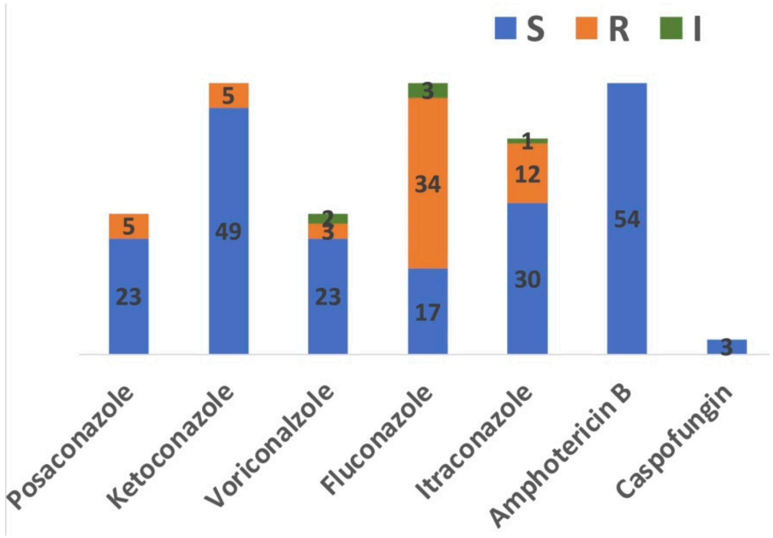

Figure S1. Antibiotic resistance pattern of clinical fungal isolates (S, Susceptible; I, Intermediate; R, Resistant).
